# Small Intestinal Bacterial Overgrowth Is a Predictor of Overt Hepatic Encephalopathy in Patients with Liver Cirrhosis

**DOI:** 10.3390/jcm14051491

**Published:** 2025-02-23

**Authors:** Akira Sakamaki, Kunihiko Yokoyama, Hanako Yamazaki, Takuya Wakabayashi, Yuichi Kojima, Kentaro Tominaga, Atsunori Tsuchiya, Kenya Kamimura, Junji Yokoyama, Shuji Terai

**Affiliations:** 1 Division of Gastroenterology and Hepatology, Graduate School of Medical and Dental Sciences, Niigata University, Niigata 951-8510, Japan; hanakoy87@outlook.jp (H.Y.); waka.8453.taku@gmail.com (T.W.); y-kojima@med.niigata-u.ac.jp (Y.K.); k-tominaga@med.niigata-u.ac.jp (K.T.); a.tsuchiya@yamanashi.ac.jp (A.T.); 2 Division of Gastroenterology and Hepatology, Niigata Prefectural Hospital, Niigata 943-0192, Japan; kunihikoyokoyama@gmail.com; 3 Department of General Medicine, Niigata University School of Medicine, Niigata 951-8510, Japan; kenya-k@med.niigata-u.ac.jp; 4 Division of Gastroenterology and Hepatology, Saiseikai Niigata Hospital, Niigata 950-1104, Japan; j.yokoyama@ngt.saiseikai.or.jp

**Keywords:** small intestinal bacterial overgrowth, hepatic encephalopathy, liver cirrhosis

## Abstract

**Objective:** Liver cirrhosis (LC) progression induces intestinal microbiota abnormalities, such as small intestinal bacterial overgrowth (SIBO), and these changes lead to the inflow of gut pathogens and their degradation products into the vessels, causing cirrhotic complications such as hepatic encephalopathy (HE). **Methods:** To clarify the relationship between the development of overt HE and SIBO, we conducted a three-year observation after assessment of SIBO in patients with LC. **Results:** In the analysis of 107 patients, with a mean follow-up duration of 29.4 months, 31 were diagnosed with SIBO and 30 with covert HE. In the Cox multivariate regression analysis for prognosis, the Child–Pugh score, blood urea nitrogen level, and the Union for International Cancer Control (UICC) stage of hepatocellular carcinoma were derived using the following five factors: white blood cell count, blood urea nitrogen level, Child–Pugh score, UICC stage, and serum aspartate aminotransferase and alkaline phosphatase levels (*p* = 0.002, hazard ratio [HR] 3.733, 95% confidence interval [CI] 1.592–8.754, *p* = 0.001, HR 1.076, 95% CI 1.030–1.123, and *p* < 0.001, HR 2.767, 95% CI 1.780–4.302, respectively). Furthermore, in the Cox multivariate regression analysis for overt HE development, covert HE and methane-producing SIBO were derived using the following four factors: methane-producing SIBO, UICC stage, covert HE, and serum ammonia levels (*p* = 0.038, HR 5.008, 95% CI 1.096–22.892 and *p* = 0.006, HR 8.597, 95% CI 1.881–39.291, respectively). **Conclusions**: M-SIBO positivity was a significant predictor of overt HE.

## 1. Introduction

Liver cirrhosis (LC), the ultimate stage of various hepatic conditions, is characterized by the presence of regenerative nodules with a fibrous septum in the whole liver [1]. Chronic viral hepatitis, steatotic liver disease, and autoimmune liver disease are common etiologies of LC. Because of recent advancements in antiviral therapy for viral hepatitis [2], the proportion of patients with LC due to steatotic liver disease, including alcoholic or metabolic-related variants, has significantly increased due to decreases in the number of those with viral hepatitis [3]. Although LC is asymptomatic during the compensated state, complications of LC with various symptoms are observed in the decompensated state. Jaundice, portal hypertension, rupture of gastroesophageal varices, ascites, spontaneous bacterial peritonitis, hepatic encephalopathy (HE), hepatorenal syndrome, and hepatopulmonary syndrome are well-known complications of LC [4].

The gut environment is altered by cirrhosis progression: small intestinal bacterial overgrowth (SIBO) [5], increased intestinal wall mucosa permeability [6], and dysfunction of the gut-associated lymphoid tissue immune system [7] occur in patients with LC. Furthermore, the interaction of these pathological gut conditions induces the inflow of gut pathogens and their degradation products, such as lipopolysaccharide, into the vessels, and causes cirrhotic complications [8]. Especially in alcohol-related and metabolic-associated steatotic liver diseases, the mechanisms of these pathological gut conditions are more important in the pathogenesis of hepatic inflammation and fibrosis [9,10].

A meta-analysis on the relationship between SIBO and LC revealed that the rate of SIBO positivity in patients with cirrhosis ranges from 34.8% to 47.1% and the odds of LC are 6.83 times higher in SIBO-positive patients. In addition, patients with decompensated cirrhosis complicated with SIBO had a higher frequency than those with compensated cirrhosis (50.5% vs. 31.2%; odds ratio: 2.56); therefore, the frequency of SIBO increases with the progression of LC [5]. We previously reported a cross-sectional study that concluded that the proportion of hydrogen producing SIBO is associated with LC progression [11]. Covert HE, hyperammonemia, sarcopenia, and spontaneous portosystemic shunts were previously identified as predictors of overt HE [12,13,14]. In addition, a transjugular intrahepatic portosystemic shunt for treating refractory ascites is also known to induce HE [15]. Therefore, we performed a three-year observation to reveal the relationship between overt HE onset and SIBO to assess the influence of the latter on overt HE complications and death.

## 2. Materials and Methods

### 2.1. Study Protocol

This was a prospective cohort study conducted at Niigata University. The study was approved by the ethical review board of Niigata University (Approval Number 2023-0209) and was performed as a follow-up study to a previous cross-sectional study [11]. We recruited participants for the study between 1 May 2019 and 31 December 2020, in patients hospitalized because of LC complications, and 121 patients agreed to participate in the study. All patients provided their written informed consent for the prospective observation at the time of case inclusion in the previous cross-sectional study. Physical assessments, including the breath test and number connection tests (NCTs), were performed after managing LC complications. Fourteen patients were excluded because of the administration of poorly absorbable antibiotics at the time of the breath test; therefore, we ended up with 107 participants in the study. After these assessments, we followed up on all 107 patients for three years. New inclusion and exclusion criteria were not established for the current study. The endpoints of the study were all-cause mortality and overt HE development.

**Figure 1:**
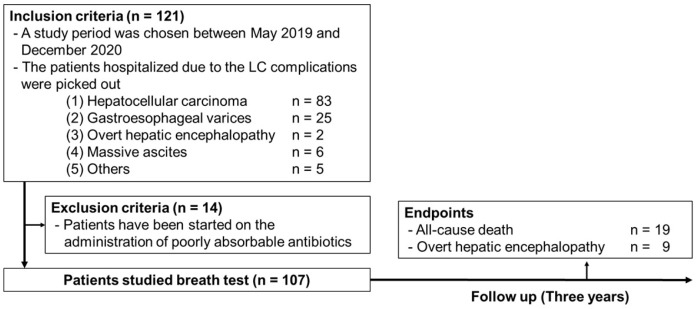
Study protocol. LC, liver cirrhosis.

This study was conducted in accordance with the principles outlined in the Declaration of Helsinki and the ethical guidelines for medical and biological research involving human subjects in Japan. The study protocol is presented in Figure 1.

### 2.2. Breath Test

The exhaled hydrogen and methane concentrations were measured using a BGA2000D (Laboratory for Expiration Biochemistry Nourishment Metabolism Co., Ltd., Nara, Japan). Fifty grams of glucose or 10 g of lactulose was used as the sugar substrate. Breath measurement was performed three times before the sugar substrate loading to determine the baseline, every 15 min after loading and up to 120 or 180 min.

The diagnostic criteria for SIBO are defined according to the North American consensus: an increase of ≥20 ppm from baseline in hydrogen by 90 min and a level of ≥10 ppm in methane [16,17]. Furthermore, patients with elevated hydrogen levels were defined as H-SIBO, whereas those with elevated methane levels were defined as M-SIBO, as previously reported [18].

### 2.3. Neuro-Psychological Tests

NCT-A and NCT-B were performed as the neuro-psychological tests. The NCTs are one of neuro-psychological tests that entails using an iPad to connect the displayed numbers and letters with the fingers for the diagnosis of covert HE. In this study, we used “Neuro-Psychological Tests (v2.1) For iPad”, available on the website accessed on 1 April 2021 (https://www.jsh.or.jp/medical/guidelines/medicalinfo/otsuka.html) provided by Otsuka Pharmaceutical Co., Ltd. Patients performed the NCT-A and NCT-B tests, and covert HE was diagnosed based on the obtention of positive results of both NCT tests [19]. The cutoff values were based on a previous report for Japanese patients [20].

### 2.4. Statistical Analysis

SPSS (version 29.0.2.0; IBM, Armonk, NY, USA) was used to perform the univariate and multivariate Cox regression analyses, and GraphPad Prism Version 8.3.0 (GraphPad Software, Inc., Boston, MA, USA) was used to generate the Kaplan–Meier survival curve.

## 3. Results

The study cohort included 81 male and 26 female patients (n = 107), with a median age of 70 (40–86) years. Hepatocellular carcinoma (HCC) occurred as a complication in 77 (72.0%) patients. Thirty-one (29.0%) patients were diagnosed with SIBO using the breath test; sixteen (15.0%) patients had increased concentrations of exhaled hydrogen, and nineteen (17.8%) had an increased concentration of exhaled methane. Four patients were found to have increased concentrations of both hydrogen and methane. When the cutoff value of hydrogen producing SIBO was defined as an increase of ≥10 ppm from baseline, 37 (34.6%) patients met that criterion, and when the cutoff value of methane producing SIBO was defined as ≥5 ppm, 24 (22.4%) patients met that criterion. Thirty (28.0%) patients were diagnosed with covert HE by the neuro-psychological tests.

Gastroesophageal varices were detected in 59 (55.1%) patients by esophagogastroduodenoscopy, and 5 (4.7%) patients had a history of gastroesophageal varices rupture. None of the patients took nonselective beta-blockers, while 24 of 59 patients received endoscopic variceal ligation, endoscopic injection sclerotherapy, or balloon-occluded retrograde transvenous obliteration as the prophylactic treatment for gastrointestinal varices according to Japanese guidelines for LC [21]. Also, none of the patients received transjugular intrahepatic portosystemic shunt or other portosystemic shunt treatment. Although no patients received liver transplantation before study inclusion, two patients (1.9%) underwent liver transplantation during the follow-up period. The mean observation period was 29.4 months in the cohort study. The characteristics of our study participants are summarized in Table 1.

First, the Cox regression analysis was performed to examine the relationship between each of the clinical characteristics and all-cause mortality. In this study, 72.0% of the patients had HCCs as mentioned above; thus, the clinical stage of HCC, as determined by the Union for International Cancer Control (UICC) or the Barcelona Clinic Liver Cancer (BCLC), was the most significant prognostic factor in the univariate analysis (*p* < 0.001, hazard ratio [HR] 2.323, 95% confidence interval [CI] 1.540–3.503, and *p* < 0.001, HR 2.259, 95% CI 1.480–3.446, respectively; Table 2). The high levels of total bilirubin (T-bil), aspartate aminotransferase (AST), blood urea nitrogen (BUN), and white blood cell (WBC) count were also associated with a poor prognosis, while the Child–Pugh grade was not significantly associated with the prognosis in the univariate analysis (*p* = 0.193, HR 1.701, 95% CI 0.764–3.790). T-bil is one of the components of the Child–Pugh score, and the Child–Pugh score is significantly associated with poor prognosis. We performed the multivariate Cox regression analysis using the following five factors: WBC, BUN, Child–Pugh score, UICC stage of HCC, and AST. As a result of the analysis, the Child–Pugh score, BUN, and UICC stage of HCC were found to be significantly associated with disease prognosis (*p* = 0.001, HR 3.733, 95% CI 1.592–8.754, *p* = 0.001, HR 1.076, 95% CI 1.030–1.123, and *p* < 0.001, HR 2.767, 95% CI 1.780–4.302, respectively; Table 2). Even if T-bil was used as a factor in the multivariate analysis, the T-bil, BUN, and UICC stage of HCC also remained the same as they were in the analysis performed using the Child–Pugh score (Table S1).

Next, Cox regression analysis was performed to examine the relationship between each of the clinical characteristics and overt HE. In the univariate analysis, M-SIBO positivity and UICC stage of HCC were identified as significantly associated factors (*p* = 0.010, HR 7.228, 95% CI 1.614–32.361 and *p* = 0.029, and HR 2.046, 95% CI 1.076–3.889, respectively; Table 3). Additionally, if the cutoff value was set at 5 ppm in methane, M-SIBO positivity would also be significantly associated with overt HE (*p* = 0.024, HR 5.649, 95% CI 1.258–25.369). While covert HE and serum ammonia levels were not significantly associated with overt HE in the univariate analysis (*p* = 0.067, HR 4.058 95% CI 0.908–18.142 and *p* = 0.079, HE 1.016 95% CI 0.998–1.035), covert HE and hyperammonemia were previously identified as predictive factors for overt HE. Therefore, the multivariate Cox regression analysis was performed using four factors: M-SIBO, UICC stage, covert HE, and serum ammonia levels, and covert HE and M-SIBO were identified in the analysis (*p* = 0.038, HR 5.008, 95% CI 1.096–22.892 and *p* = 0.006, HR 8.597, 95% CI 1.881–39.291, respectively; Table 3), even if cutoff value was set at 5 ppm in methane for the diagnosis of M-SIBO (*p* = 0.027, HR 5.668, 95% CI 1.222–26.29, and *p* = 0.010, HR 7.663, 95% CI 1.639–35.832, respectively; Table S2). Furthermore, the multivariate analysis was performed using only two factors (M-SIBO and the UICC stage), which were significantly associated with overt HE in the univariate analysis, and only M-SIBO emerged as an independently associated factor in this analysis (Table S3).

**Figure 2:**
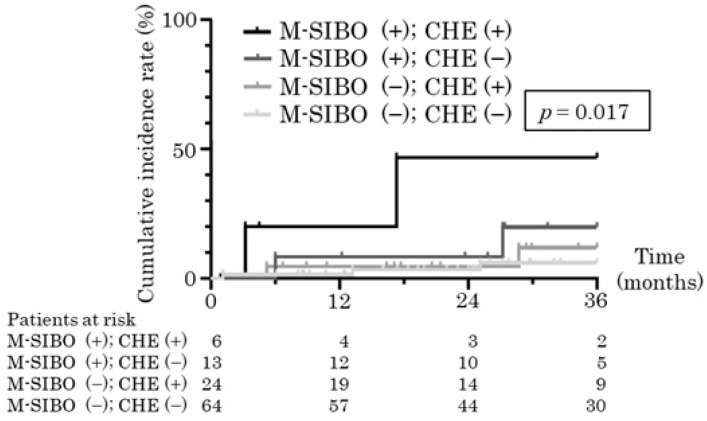
Cumulative incidence curve of overt hepatic encephalopathy in at-risk patients. The patients were divided into four groups according to the presence or absence of covert HE and M-SIBO. The group of patients with neither covert HE nor M-SIBO had a cumulative three-year incidence rate of 5.9%, the group with covert HE without M-SIBO was 11.9%, the group with M-SIBO without covert HE was 19.8%, and the group of patients with both covert HE and M-SIBO was 46.7% (*p* = 0.017). M-SIBO, methane-producing small intestinal bacterial overgrowth; CHE, covert hepatic encephalopathy.

Based on the results of the multiple Cox regression analysis, the patients were divided into four groups according to the presence or absence of covert HE and M-SIBO, and a cumulative incidence analysis was performed. The group of patients with neither covert HE nor M-SIBO had a cumulative three-year incidence rate of 5.9%, compared with 46.7% in the group of patients with both covert HE and M-SIBO (*p* = 0.017, Figure 2). Per our findings, both M-SIBO and covert HE are risk factors for overt HE development.

## 4. Discussion

In a previous cross-sectional study, we reported that H-SIBO was significantly associated with LC progression and covert HE [11]. Additionally, a meta-analysis reported that patients with decompensated cirrhosis exhibited SIBO complications with higher frequency than the patients with compensated cirrhosis [5]. In detail, only H-SIBO was measured in the meta-analysis, and it determined the presence of SIBO. Based on the results of the meta-analysis and our previous study, both the frequency of covert HE and the H-SIBO complication rate increased proportionately with LC progression. As a result, a statistically significant association was observed between H-SIBO and covert HE.

Although there is no gold standard for the diagnosis of SIBO, two diagnostic methods have been reported. First, proximal jejunal fluid culture was used as a direct diagnostic method for SIBO, but this method has several limitations: the invasiveness of proximal jejunal fluid collection, the presence of nonculturable bacteria, and the possibility of contamination with oral bacteria [16]. Second, the breath test was used as an indirect method. The mechanism of the breath test is that the orally ingested sugar substrates are metabolized by the intestinal bacteria, and the metabolites are detected in the exhaled breath. Because of its simplicity and low invasiveness, the breath test is used worldwide to detect SIBO [5]. However, the interpretation of breath tests is also fraught with controversy. In diagnosing SIBO using the hydrogen breath test, a cutoff value of 20 ppm is reported to have a sensitivity of 60% and specificity of 100%, whereas a cutoff value of 10 ppm has a sensitivity of 70% and specificity of 92% [22]. Furthermore, in diagnosing methane producing SIBO, a cutoff value of 10 ppm is reported to have a sensitivity of 86.4% and specificity of 100%, whereas a cutoff value of 5 ppm has a sensitivity of 96.1% and specificity of 99.7% [17]. Based on the results, the North American Consensus recommend a diagnosis of SIBO in the case of an increase of ≥20 ppm from baseline in hydrogen by 90 min and a level of ≥10 ppm in methane [17]. Therefore, we also used a hydrogen cutoff value of 20 ppm and a methane cutoff value of 10 ppm in this study, but its low sensitivity may have caused some SIBO cases to be missed. In fact, if the cutoff value is set at 10 ppm, the number of H-SIBO cases increases from 16 to 37. However, the use of these cutoff values did not result in any difference in the results of the Cox analysis in this study (Table 2 and Table 3).

In the present observational study, H-SIBO had no significant relationship with the incidence of overt HE, whereas M-SIBO was a predictor of overt HE. Methane has been reported to decrease intestinal transit velocity in animal studies [23] and to be associated with constipation in humans, with the severity of constipation correlating with the methane concentration [24,25]. It is well-known that constipation is one of the triggers of overt HE [26]; therefore, M-SIBO-positive patients with LC are considered more constipated and more prone to encephalopathy than M-SIBO-negative patients. M-SIBO-positive patients exist regardless of their hepatic function; therefore, they are considered to have the potential to develop overt HE when their liver function deteriorates.

In addition, it is already known that LC and portal hypertension cause intestinal motility disorders [27,28]. In patients with LC, the decrease in motility of the small intestine and the prolongation of the small intestinal transit time cause the development of SIBO. Therefore, impaired small intestinal motility is associated with SIBO and the development of HE, and there may be no direct causal relationship between SIBO and overt HE. To assess this direct relationship, further detailed investigation, including with regard to gastrointestinal function, is expected in the future.

The key drugs for the treatment of overt HE and the prevention of recurrence in patients with LC are synthetic disaccharides and poorly absorbable oral antibiotics. Synthetic disaccharides, represented by lactulose, are expected to have a laxative effect and improve the gut microbiome and flora [29,30]. Synthetic disaccharides have been reported to improve not only HE, but also both liver-related and overall mortality rates in patients with HE in a systematic review [31], and the agents are the first choice of treatment for overt HE in the American Association for the Study of Liver Disease, the European Association for the Study of the Liver, and the Japan Society of Hepatology guidelines [21,32,33]. On the other hand, poorly absorbable oral antibiotics, such as rifaximin, are expected to inhibit ammonia-producing bacteria in the gut [34]. Rifaximin has been reported to prevent HE recurrence in a systematic review and may also improve the HE recovery and mortality rates [35]. Poorly absorbable oral antibiotics are also recommended by the guideline to use for patients with HE, the same as lactulose [21].

Rifaximin is also used in the treatment of SIBO, and its usefulness has already been reported with the improved rate of treatment with rifaximin for SIBO being 70% [36]. However, to the best of our knowledge, no reports were found that lactulose was administered for therapy in patients with SIBO in our literature survey. Therefore, for the treatment of patients with H-SIBO complicated by cirrhosis, poorly absorbable oral antibiotics may be more suitable. *Methanobrevibacter smithii*, the predominant methanogenic bacterium in the human gut, is resistant to many antibiotics; therefore, antibiotics are not considered effective for the treatment of M-SIBO [37,38,39]. Furthermore, lactulose is frequently used to treat constipation. Therefore, for the treatment of patients with H-SIBO complicated by cirrhosis, synthetic disaccharides may be more suitable. In addition, serum ammonia levels and neuro-psychological tests are considered to be performed more frequently to prevent the complication of overt HE in patients with M-SIBO.

This study has some limitations. The study protocol had a small sample size and was performed in a single center; thus, the risk of regional bias and statistical error is undeniable. A multicenter study with a larger number of patients is needed for the results to be generally applicable.

## 5. Conclusions

M-SIBO positivity was a significant predictor of overt HE. In patients with M-SIBO, careful follow-up is needed to avoid complicating overt HE in patients with cirrhosis. While there is no evidence regarding drug selection for patients with SIBO and HE, and further studies are needed, poorly absorbable oral antibiotics for H-SIBO and synthetic disaccharides for M-SIBO may be more suitable when considering the pathophysiology of each SIBO.

## Figures and Tables

**Table 1:** Characteristics of our study participants.

Median (Min–Max)		Median (Min–Max)	
or n (%)	n = 107	or n (%)	n = 107
Age, years	70 (40–86)	Albumin, g/dL	3.8 (2.1–5.0)
Gender		Total bilirubin, mg/dL	0.9 (0.3–14.2)
Males	81 (75.7)	Prothrombin time, %	92 (25–131)
Females	26 (24.3)	Ammonia, μg/dL	64 (28–242)
Body mass index, kg/m^2^	24.8 (12.6–47.7)	Creatinine, mg/dL	0.81 (0.45–3.31)
The etiology of liver cirrhosis		Blood urea nitrogen, mg/dL	16 (5–61)
Hepatitis B virus	20 (18.7)	White blood cell count, ×10^3^/µL	4.3 (1.3–14.8)
Hepatitis C virus	23 (21.5)	Platelet count, ×10^4^/µL	10.9 (2.6–26.3)
Alcoholic liver disease	31 (29.0)	Child–Pugh score	5 (5–12)
Non-alcoholic steatohepatitis	23 (21.5)	Child–Pugh grade (A/B/C)	85/17/5
Others	10 (9.3)	ALBI score	−2.45 (−0.47–−3.32)
PPI administration	63 (58.9)	mALBI grade (1/2a/2b/3)	38/29/31/9
Gastroprokinetic drug administration	6 (5.6)	Gastroesophageal varices	59 (55.1)
Probiotics administration	14 (13.1)	Rupture of gastroesophageal varices	5 (4.7)
HCC complication	77 (72.0)	Covert HE	30 (28.0)
UICC stage of HCC (I/II/III/IV)	18/29/17/13	SIBO	31 (29.0)
BCLC stage of HCC (I/II/III/IV)	30/31/14/2	Hydrogen producing SIBO	16 (15.0)
Aspartate aminotransferase, U/L	36 (12–209)	Methane producing SIBO	19 (17.8)
Alanine aminotransferase, U/L	29 (10–217)	An increase of ≥10 ppm in hydrogen	37 (34.6)
Alkaline Phosphatase, U/L	281 (64–3147)	≥5 ppm in methane	24 (22.4)
Gamma-glutamyl transpeptidase, U/L	68 (13–691)	Observation period, m	29.4 (0.9–36.0)
Cholinesterase, U/L	210 (47–484)		

**Table 2:** Cox regression analyses for the patient’s prognosis.

	Univariate Analysis		Multivariate Analysis	
	*p* Value	Hazard Ratio	*p* Value	Hazard Ratio
Age, years	0.948	1.002 (0.956–1.049)		
Gender	0.210	0.392 (0.090–1.696)		
Body mass index, kg/m^2^	0.431	1.031 (0.955–1.114)		
The etiology of liver cirrhosis				
Hepatitis B virus				
Hepatitis C virus				
Alcoholic liver disease				
Non-alcoholic steatohepatitis				
Others				
PPI administration	0.109	0.474 (0.191–1.181)		
HCC complication	0.102	3.399 (0.785–14.721)		
UICC stage of HCC (0/I/II/III/IV)	<0.001 *	2.323 (1.540–3.503)	<0.001 *	2.767 (1.780–4.302)
BCLC stage of HCC (0/I/II/III/IV)	<0.001 *	2.259 (1.480–3.446)		
Aspartate aminotransferase, U/L	0.037 *	1.013 (1.001–1.025)	0.074	
Alanine aminotransferase, U/L	0.146	1.013 (0.996–1.030)		
Alkaline Phosphatase, U/L	0.008 *	1.001 (1.000–1.003)	0.402	
Gamma-glutamyl transpeptidase, U/L	0.066	1.003 (1.000–1.006)		
Cholinesterase, U/L	0.669	1.001 (0.995–1.008)		
Albumin, g/dL	0.424	0.712 (0.309–1.639)		
Total bilirubin, mg/dL	0.025 *	1.247 (1.028–1.513)		
Prothrombin time, %	0.768	1.004 (0.979–1.030)		
Ammonia, μg/dL	0.348	1.006 (0.993–1.020)		
Creatinine, mg/dL	0.316	1.587 (0.643–3.914)		
Blood urea nitrogen, mg/dL	0.009 *	1.047 (1.011–1.084)	0.001 *	1.076 (1.030–1.123)
White blood cell count, ×10^3^/µL	0.021 *	1.193 (1.027–1.385)	0.626	
Platelet count, ×10^4^/µL	0.173	1.052 (0.978–1.131)		
Child–Pugh score	0.471	1.117 (0.827–1.508)	0.002 *	3.733 (1.592–8.754)
Child–Pugh grade (A/B/C)	0.193	1.701 (0.764–3.790)		
ALBI score	0.392	1.448 (0.620–3.381)		
mALBI grade (1/2a/2b/3)	0.732	1.086 (0.676–1.747)		
Gastroesophageal varices Rupture of gastroesophageal varices	0.085 0.538	0.441 (0.173–1.120) 0.046 (0.000–808.7)		
Covert HE	0.479	1.418 (0.539–3.733)		
SIBO	0.483	1.414 (0.537–3.721)		
Hydrogen producing SIBO	0.130	2.348 (0.777–7.096)		
Methane producing SIBO An increase of ≥10 ppm in hydrogen	0.983 0.996	1.013 (0.295–3.479) 1.003 (0.381–2.639)		
≥5 ppm in methane	0.898	1.075 (0.356–3.242)		

PPI, proton pump inhibitor; HCC, hepatocellular carcinoma; UICC, Union for International Cancer Control; BCLC, Barcelona Clinic Liver Cancer; ALBI, Albumin–Bilirubin; mALBI, modified ALBI; HE, hepatic encephalopathy; SIBO, small intestinal bacterial overgrowth. *: *p* value < 0.05.

**Table 3:** Cox regression analyses for the onset of overt hepatic encephalopathy.

	Univariate Analysis		Multivariate Analysis	
	*p* Value	Hazard Ratio	*p* Value	Hazard Ratio
Age, years	0.631	0.982 (0.914–1.056)		
Gender	0.218	2.562 (0.573–11.458)		
Body mass index, kg/m^2^	0.159	1.079 (0.971–1.199)		
The etiology of liver cirrhosis				
Hepatitis B virus				
Hepatitis C virus				
Alcoholic liver disease				
Non-alcoholic steatohepatitis				
Others				
PPI administration	0.310	0.460 (0.102–2.062)		
HCC complication	0.406	2.455 (0.295–20.423)		
UICC stage of HCC (0/I/II/III/IV)	0.029 *	2.046 (1.076–3.889)	0.065	
BCLC stage of HCC (0/I/II/III/IV)	0.088	1.804 (0.917–3.551)		
Aspartate aminotransferase, U/L	0.065	1.017 (0.999–1.035)		
Alanine aminotransferase, U/L	0.587	1.009 (0.977–1.041)		
Alkaline Phosphatase, U/L	0.567	1.001 (0.998–1.003)		
Gamma-glutamyl transpeptidase, U/L	0.052	1.004 (1.000–1.008)		
Cholinesterase, U/L	0.073	0.989 (0.978–1.001)		
Albumin, g/dL	0.178	0.443 (0.135–1.449)		
Total bilirubin, mg/dL	0.189	1.202 (0.913–1.581)		
Prothrombin time, %	0.406	0.985 (0.949–1.021)		
Ammonia, μg/dL	0.079	1.016 (0.998–1.035)	0.145	
Creatinine, mg/dL	0.476	1.719 (0.387–7.632)		
Blood urea nitrogen, mg/dL	0.338	1.036 (0.964–1.114)		
White blood cell count, ×10^3^/µL	0.391	0.804 (0.488–1.324)		
Platelet count, ×10^4^/µL	0.443	0.940 (0.803–1.100)		
Child–Pugh score	0.148	1.328 (0.904–1.952)		
Child–Pugh grade (A/B/C)	0.165	2.270 (0.714–7.221)		
ALBI score	0.109	2.625 (0.806–8.551)		
mALBI grade (1/2a/2b/3)	0.129	1.826 (0.840–3.970)		
Gastroesophageal varices Rupture of gastroesophageal varices	0.451 0.741	1.878 (0.364–9.686) 0.047 (0.000–3.61 × 10^6^)		
Covert HE	0.067	4.058 (0.908–18.142)	0.038 *	5.008 (1.096–22.892)
SIBO	0.015 *	7.610 (1.475–39.625)		
Hydrogen producing SIBO	0.131	3.553 (0.685–18.415)		
Methane producing SIBO An increase of ≥10 ppm in hydrogen	0.010 * 0.550	7.228 (1.614–32.361) 1.579 (0.353–7.060)	0.006 *	8.597 (1.881–39.291)
≥5 ppm in methane	0.024 *	5.649 (1.258–25.369)		

HE, hepatic encephalopathy; PPI, proton pump inhibitor; HCC, hepatocellular carcinoma; UICC, Union for International Cancer Control; BCLC, Barcelona Clinic Liver Cancer; ALBI, Albumin-Bilirubin; mALBI, modified ALBI; HE, hepatic encephalopathy; SIBO, small intestinal bacterial overgrowth. *: *p* value < 0.05.

## Data Availability

The original contributions presented in this study are included in the article/Supplementary Materials. Further inquiries can be directed to the corresponding authors.

## Supplementary Materials

The following supporting information can be downloaded at https://www.mdpi.com/article/10.3390/jcm14051491/s1, Table S1: Cox regression analyses for the patient’s prognosis using total bilirubin levels; Table S2: Cox regression analyses for the onset of overt hepatic encephalopathy with the cutoff value of 5 ppm in methane; Table S3: Cox regression analyses for the onset of overt hepatic encephalopathy without serum ammonia levels and covert hepatic encephalopathy.

## Abbreviations

The following abbreviations are used in this manuscript:

LC	Liver cirrhosis
SIBO	Small intestinal bacterial overgrowth
HE	Hepatic encephalopathy
H-SIBO	Hydrogen producing SIBO
M-SIBO	Methane producing SIBO
NCT	Number connection test
HCC	Hepatocellular carcinoma
UICC	Union for International Cancer Control
BCLC	Barcelona Clinic Liver Cancer
HR	Hazard ratio
CI	Confidence interval
T-bil	Total bilirubin
AST	Aspartate aminotransferase
BUN	Blood urea nitrogen
WBC	White blood cell
