# Supplementary material for: Small Intestinal Bacterial Overgrowth Is a Predictor of Overt Hepatic Encephalopathy in Patients with Liver Cirrhosis
Source: J Clin Med. 2025 Feb 23;14(5):1491. doi: 10.3390/jcm14051491 (PMC11901010; doi:10.3390/jcm14051491)
Supplement: Supplementary file 1 [file jcm-14-01491-s001.zip › jcm-3440609-supplementary.pdf]

Table S1. Cox regression analyses for the patient's prognosis using total bilirubin levels.

	univariate analysis		multivariate analysis	
	p value	hazard ratio	p value	hazard ratio
UICC stage of HCC (0/I/II/III/IV)	**< 0.001***	**2.323 (1.540 – 3.503)**	**< 0.001***	**2.420 (1.570 – 3.728)**
Aspartate aminotransferase, U/L	**0.037***	**1.013 (1.001 – 1.025)**	0.055	
Alkaline Phosphatase, U/L	**0.008***	**1.001 (1.000 – 1.003)**	0.201	
Total bilirubin, mg/dL	**0.025***	**1.247 (1.028 – 1.513)**	**0.022***	**1.219 (1.029 – 1.442)**
Blood urea nitrogen, mg/dL	**0.009***	**1.047 (1.011 – 1.084)**	**0.002***	**1.070 (1.026 – 1.116)**
White blood cell count, x10^3/μL	**0.021***	**1.193 (1.027 – 1.385)**	0.905	

UICC, Union for International Cancer Control; HCC, hepatocellular carcinoma. *: P value < 0.05.

Table S2. Cox regression analyses for the onset of overt hepatic encephalopathy with the cutoff value of 5 ppm in methane.

	univariate analysis		multivariate analysis	
	p value	hazard ratio	p value	hazard ratio
UICC stage of HCC (0/I/II/III/IV)	**0.029***	**2.046 (1.076 – 3.889)**	0.052	
Ammonia, μg/dL	0.079	1.016 (0.998 – 1.035)	0.121	
Covert HE	0.067	4.058 (0.908 – 18.142)	**0.027***	**5.668 (1.222 – 26.29)**
≥5 ppm in methane	**0.024***	**5.649 (1.258 – 25.369)**	**0.010***	**7.663 (1.639 – 35.832)**

UICC, Union for International Cancer Control; HCC, hepatocellular carcinoma. *: P value < 0.05.

Table S3. Cox regression analyses for the onset of overt hepatic encephalopathy without serum ammonia levels and covert hepatic encephalopathy.

	univariate analysis		multivariate analysis	
	p value	hazard ratio	p value	hazard ratio
UICC stage of HCC (0/I/II/III/IV)	**0.029***	**2.046 (1.076 – 3.889)**	0.057	
Methane producing SIBO	**0.010***	**7.228 (1.614 – 32.361)**	**0.010***	**7.228 (1.614 – 32.361)**

UICC, Union for International Cancer Control; HCC, hepatocellular carcinoma; SIBO, small intestinal bacterial overgrowth. *: P value < 0.05.
